# Changes in psychosocial functioning among urban, school-age children during the COVID-19 pandemic

**DOI:** 10.1186/s13034-021-00419-w

**Published:** 2021-12-02

**Authors:** Andrea E. Spencer, Rachel Oblath, Rohan Dayal, J. Krystel Loubeau, Julia Lejeune, Jennifer Sikov, Meera Savage, Catalina Posse, Sonal Jain, Nicole Zolli, Tithi D. Baul, Valeria Ladino, Chelsea Ji, Jessica Kabrt, Lillian Mousad, Megan Rabin, J. Michael Murphy, Arvin Garg

**Affiliations:** 1 Department of Psychiatry, Boston University School of Medicine, Boston Medical Center, Boston, MA 02118 USA; 2 Department of Psychiatry, Massachusetts General Hospital, Harvard Medical School, Boston, MA USA; 3 Department of Pediatrics, University of Massachusetts Medical School, Worcester, MA USA

**Keywords:** COVID-19, Child psychiatry, Social determinants of health, Urban health, Minority health, Anxiety, Depression

## Abstract

**Background:**

There is concern about the effect of the COVID-19 pandemic on psychosocial functioning among school-age children, who have faced unusual stressors during this time. Our goal was to assess mental health symptoms and social risks during COVID-19, compared to before the pandemic, for urban, racial and ethnic minority school-age children, and investigate the relationship between mental health and social risks.

**Methods:**

We conducted a cohort study from September 2019 until January 2021 of children age 5–11 years old recruited from an urban safety net hospital-based pediatric primary care practice. We measured emotional and behavioral symptoms (including attention, internalizing, and externalizing symptoms) before and during the pandemic with the Pediatric Symptom Checklist (PSC-17). We measured social risks (including food and housing insecurity) before and during the pandemic with the THRIVE screener. We measured additional mid-pandemic COVID-related stressors with items on school participation, screens/media use, illness exposure, and caregiver mental health. We compared pre- and mid-pandemic PSC-17 symptom scores across 4 domains (total, attention, internalizing, and externalizing) and used path analysis to examine the relationship between mental health and social risks pre- and mid-pandemic.

**Results:**

Caregivers of 168 children (54% non-Hispanic Black, 29% Hispanic, and 22% non-English speaking) completed the study. Children had significantly higher levels of emotional and behavioral symptoms midpandemic- vs. pre-pandemic in all domains. Significantly more children had a positive PSC-17 total score (18% vs. 8%, p < 0.01) and internalizing (depression and anxiety) score (18% vs. 5%, p < 0.001) during the pandemic vs. before, indicating clinical concerns in these areas. Caregivers reported significantly more social risks during vs. before the pandemic (p < 0.001). Mental health symptoms significantly correlated with number of social risks before the pandemic, but not during the pandemic. Less school assignment completion, increased screen time, and caregiver depression were all significantly associated with worse mid-pandemic mental health in children.

**Conclusion:**

The COVID-19 pandemic has led to a dramatic increase in depression/anxiety problems and social risks among urban, racial and ethnic minority school-age children compared to before the pandemic. More research is needed to understand if these changes will persist.

**Supplementary Information:**

The online version contains supplementary material available at 10.1186/s13034-021-00419-w.

## Introduction

Since COVID-19 was declared a global pandemic, [1] children have faced many pandemic-related adversities, including social isolation, school closures, increased screen time, stressed caregivers, financial difficulties, reduced access to health care, and loss of loved ones [2]. One year later, experts fear that this pandemic may have led to another public health crisis: a surge in psychosocial problems among children [3]. Amid growing concern even pre-pandemic about meeting the needs of children with mental health problems in the US, [4] worsening child mental health could lead to a mental health crisis for an entire generation, [5, 6] including lifelong adverse consequences and increased rates of suicide.

Given the associations between adverse childhood events, poverty, and mental health, there is particular concern for racial and ethnic minority youth, who are likely to be disproportionately impacted by pandemic-related stressors and psychosocial problems [7–11]. Racial and ethnic minority youth experience disproportionate burden of psychosocial risks associated with chronic, impairing mental health problems [12]. These include poverty and unmet social needs such as food insecurity, which pre-COVID was more than twice as common in Hispanic and non-Hispanic Black households compared to white households in the US [13]. Food insecurity is associated with a range of psychiatric problems in children and adolescents, including inattention/hyperactivity, [14] aggression/anxiety, [15] and dramatically higher rates of suicide attempts [16]. Other community factors including violence exposure and discrimination contribute to worse mental health for racial and ethnic minority youth [12, 17]. Despite experiencing many risk factors for psychopathology at high rates, racial and ethnic minority children are less likely to receive mental health treatment [12, 18]. Maternal depression, which also dramatically increases a child's risk for psychopathology, is also considerably more prevalent in racial and ethnic minority youth, [12, 19] but Black and Latina mothers are less likely to receive treatment for postpartum depression [20]. Many of these psychosocial stressors including poverty and food insecurity, community violence, and difficult access to care all worsened during the COVID-19 pandemic, particularly for urban, racial and ethnic minority children, raising particular concern about the mental health of this group of children during the pandemic.

A number of studies have begun to illustrate the impact of the pandemic on child mental health, with most concluding that child mental health had worsened during the pandemic [21–25]. The most commonly reported symptoms included depression and anxiety, with very high prevalence estimates in a few studies (e.g., 50% for depression and over 60% for anxiety) [22]. Existing literature suggests that repeated exposure to COVID-19 media coverage, increased social media use, and having a family member working on the pandemic front line have contributed to these mental health impacts, which are reported most profoundly in adolescent females [22]. However, there are key gaps that exist in the literature on child mental health during the COVID-19 pandemic. First, the vast majority of studies are cross-sectional, without pre-pandemic data to compare to mid-pandemic samples. This limits interpretation of most studies, which rely on regional or national estimates as comparators. Second, most studies have been conducted with adolescents as opposed to preadolescent children. Finally, very few studies have been conducted with populations who are most susceptible to the impacts of COVID-19, including low-income populations and minoritized populations in the US. One study raised the concern that low-income, preadolescent children might be particularly high risk for mental health deterioration from the COVID-19 pandemic [26]. We found no studies that measured the mental health impacts of acute rises in financial hardship and unmet basic needs such as food insecurity on child mental health. In addition, there is some conflicting data showing that particularly for younger, pre-adolescent children, some may actually have experienced improved mental health during the COVID-19 pandemic. For example, one study using the 19-item, Brief Problem Monitor screening tool to measure mental health in US children before and during the COVID-19 pandemic showed improved overall mental health among Hispanic youth age 10–14 years old, who were recruited from one charter school in Texas between January and May of 2020 [27]. Other studies have reported that some younger children have felt calmer or experienced improved symptoms during the pandemic, and have enjoyed spending more quality time with family [22]. Thus, there is a need for more studies measuring psychosocial symptoms before and after the pandemic among preadolescent, school-age children—particularly those who have the most exposure to pandemic-related hardship—in order to best evaluate the scope of this new public health concern.

The goal of the current study was to compare mental health symptoms before and during the pandemic, in a sample of racially and ethnically diverse school-aged children from an urban, safety-net, hospital-based pediatric primary care practice. We examined changes in the severity of overall emotional and behavioral symptoms, as well as changes within specific symptom domains (internalizing, externalizing, and attention). We also sought to understand the relationship between child mental health and social risks (e.g., food insecurity and difficulty paying bills) during the pandemic, including pandemic-specific stressors (e.g., school assignment completion, screen time). We hypothesized that more children would have mental health problems during the pandemic than before, and that worse mental health would be associated with increased social risks.

## Methods

### Research design and setting

We conducted a cohort study with children who had been screened for emotional and behavioral symptoms and social risks as part of routine care at an urban, safety-net, hospital-based, pediatric primary care clinic in the 6 months before pandemic onset. Screened children were between 5 and 11 years old at the time of their pre-pandemic well visit. We contacted legal guardians (“caregivers” hereafter) to re-administer the same mental health and social risk screeners, adding a brief questionnaire of other COVID-specific variables, three times at 3-month intervals. Here, we present results comparing data from the pre-pandemic clinical visit (conducted between September 2019 and February 2020) to the initial mid-pandemic assessment (conducted between August 2020 and January 2021).

We identified potentially eligible children using electronic health records (EHRs). Eligible children had attended well visits between September 1, 2019 and March 1, 2020, at which their caregivers had completed a universal emotional and behavioral symptom screener, the Pediatric Symptom Checklist (PSC-17) [28]. Children were excluded if complete PSC-17 results were not documented; if their caregiver could not understand informed consent procedures in English, Spanish, or Haitian Creole; or if their sibling had already participated (to enroll unique households).

From August 2020 until January 2021, trained research staff fluent in the preferred language of each household contacted caregivers by phone in a randomized order. For caregivers interested in participating, informed consent procedures were conducted by phone and an electronic informed consent document was sent and signed via email or text via the electronic, HIPAA-compliant database, REDCap [29, 30]. Research staff then sent a REDCap questionnaire via email or text to caregivers in the language of their choice (English, Spanish, or Haitian Creole). Research staff also offered to read questions to participants if preferred. When applicable, caregivers were instructed to complete the questionnaire only for the first child in randomized order. Caregivers received a $20 gift card at each time point for participating.

The study was approved by the Boston University Medical Campus Institutional Review Board.

### Measures

*Emotional and behavioral symptoms* The Pediatric Symptom Checklist (PSC-17) is used for routine mental health screening at our study site in six languages including Spanish and Haitian Creole, and item-level pre-pandemic PSC-17 results were obtained from EHRs. Caregivers complete the PSC-17 on paper, rating a series of 17 child symptoms on a three-point Likert scale based on how often they are present (*never* = *0, sometimes* = *1*, or *often* = *2*). Responses are summed to produce a total problems score and three subscale scores: internalizing (depression and anxiety) problems, externalizing (behavioral) problems, and attention problems. Higher scores indicate more severe symptoms, and each subscale also has a cut-off indicative of clinically concerning symptoms in that domain. The PSC-17 was developed via factor analysis from the longer, 35-item Pediatric Symptom Checklist (PSC-35), and studies have shown it is valid and reliable in detecting children at high mental health risk overall and in each domain [28, 31, 32]. In a recent, updated validation study of the PSC-17 with a sample of 80,680 children, [28] Cronbach’s α was 0.87 for the overall PSC-17, 0.78 for the internalizing subscale, 0.82 for attention, and 0.80 for externalizing—all very similar to the original derivation study [33]. Test–retest reliability was also good (ICC = 0.85) [28, 33]. In our sample, which included English, Spanish, and Haitian Creole speakers, the overall PSC-17 demonstrated strong internal consistency, with Cronbach’s α coefficients of 0.88 pre-pandemic and 0.90 mid-pandemic. On the three subscales, pre- and mid-pandemic α’s were 0.75 and 0.83 for internalizing; 0.86 and 0.82 for attention; and 0.81 at both time points for externalizing.

*Social Risks* The THRIVE screening tool is a questionnaire developed at our hospital that evaluates families’ social risks and unmet needs [34]. THRIVE was developed as an adaptation of WE CARE (Well Child Care, Evaluation, Community Resources, Advocacy, Referral, Education), a pediatric social needs screening and referral program [35]. THRIVE includes the “hunger vital sign,” a validated 2-item food insecurity screener, [36] as well as items on housing instability, unemployment, medication affordability, transportation to medical appointments, educational goals, and the ability to pay bills and meet family caregiving needs [34]. THRIVE is administered routinely at in-person pediatric visits at our study site, and item-level pre-pandemic data was obtained from EHRs. We examined individual social risks and also tallied the number of risks per family to make a social risk “score,” which has been shown to correlate with mental health problems in school-age children [7].

*Caregiver Mental Health* We assessed mid-pandemic caregiver symptoms of depression and anxiety using the Patient Health Questionnaire-2 (PHQ-2) [37] and General Anxiety Disorder-2 (GAD-2) [38]. Both of these questionnaires, which each have two items answered along a 4-point Likert scale, were developed as more efficient screening counterparts to their longer versions, the PHQ-9 [39] and the GAD-7 [40]. In the original validation study, a score of ≥ 3 on the PHQ-2 had a sensitivity of 83% and specificity of 92% for major depression compared to structured diagnostic interview with a mental health professional [37]. Receiver operating characteristic analysis also identified 3 as the optimal cutpoint for use as a screen, and increasing PHQ-2 scores from 0 to 6 were associated with increasing functional impairment. In a meta-analysis on validation studies of the GAD-2, pooled sensitivity and specificity from six samples showed a sensitivity of 0.76 and specificity of 0.81 for generalized anxiety disorder at a cutpoint of 3, [38] In our study, Cronbach’s α was 0.81 for the PHQ-2 and 0.86 for the GAD-2, both collected mid-pandemic only.

*Additional mid-pandemic variables* The research team created a brief questionnaire to obtain mid-pandemic information about stressors we hypothesized would be associated with child mental health during the pandemic, including completion of remote school assignments, screen time usage and change during the pandemic, exposure to COVID-related media, and knowing someone who contracted COVID-19. Items were developed and ultimately approved by the first author and both senior authors, who are experts in child psychiatry (AS), pediatrics (AG), and pediatric psychology and survey development (JMM), following review of existing questionnaires and discussion of authors’ clinical experience during the initial months of the pandemic. The goal was to develop only a few items that would provide some information on stressors of primary concern to the research team without dramatically increasing respondent burden. We also included the following open-ended item: “Please tell us anything else you think is important about how coronavirus has impacted your child’s health or well-being.” These additional mid-pandemic questions are available as Additional file 1.

*Sociodemographic variables* We extracted the following sociodemographic data from the EHR: child's age, gender, race, ethnicity, health insurance status (*public, private*, or *uninsured*), and caregivers’ preferred language.

*Needs Assistance* At the end of the survey, we asked parents if they would like assistance with any resources or referrals from research staff. Parents who answered “yes” could write in (free text) what they needed help with, and received a follow up call from research staff to further discuss and help when possible. Research staff tracked the category of need and whether they were able to help meet the need requested.

### Data analysis

Data analysis was conducted in Stata 16.1 [41]. We used paired sample t-tests to compare pre- and mid-pandemic PSC-17 scores, as well as pre- and mid-pandemic THRIVE. Cohen’s *d* was used to compute effect size (small = 0.2, medium = 0.5, large = 0.8). Two-sample tests of proportions were used to compare pre- and mid-pandemic rates for positive PSC-17 total and subscale scores and for individual social risks.

**Fig. 1:**
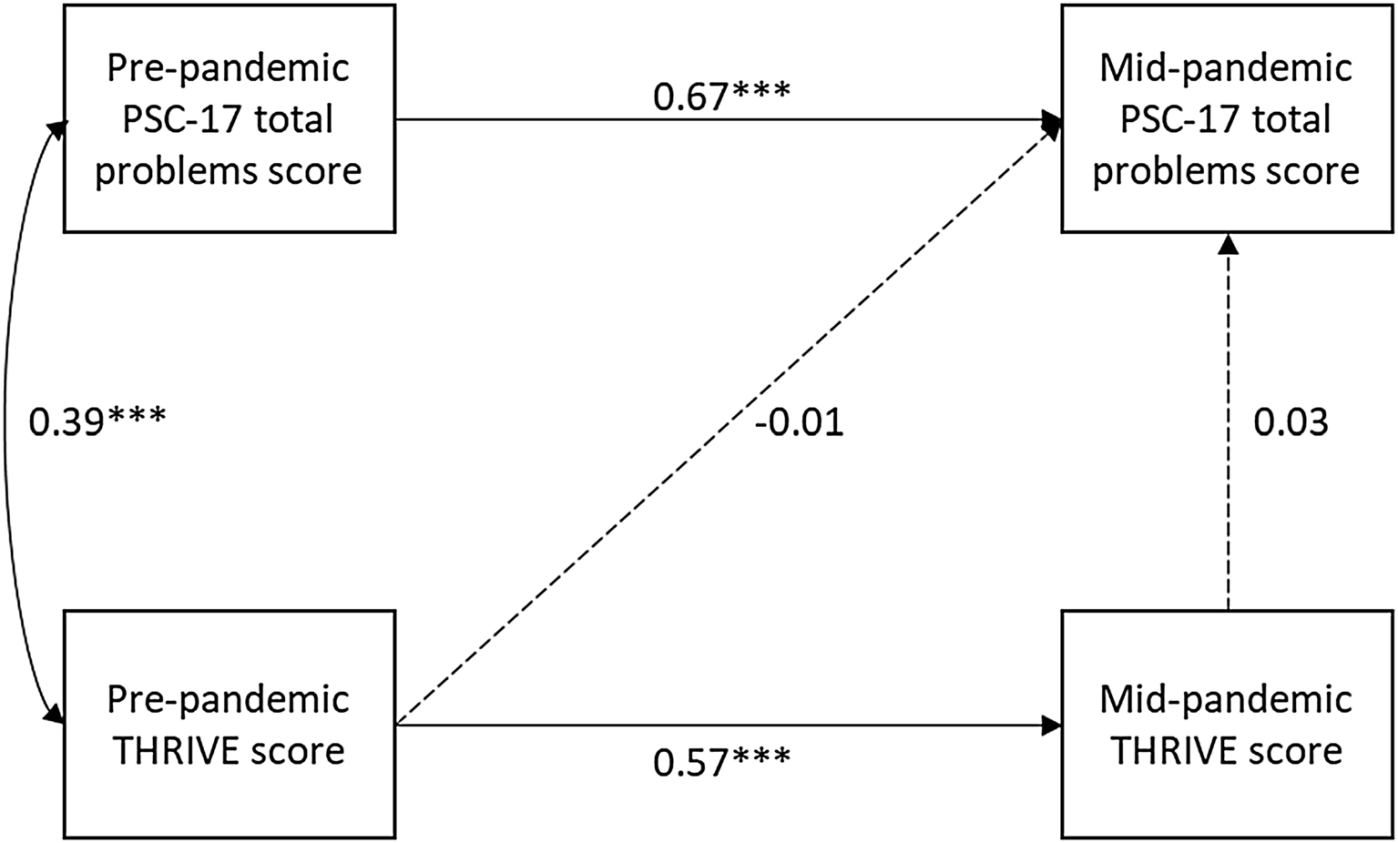
Path Model: Child Emotional and Behavioral Symptoms (PSC-17) and Social Risks (THRIVE) Before and During the Pandemic. *p < 0.05, **p < 0.01, ***p < 0.001. *PSC-17* 17-item Pediatric Symptom Checklist, *THRIVE* Social Risks Screening Tool

Path analysis with maximum likelihood estimation was used to explore the hypothesized relationships between the PSC-17 total score and number of social risks both before and during the pandemic (Fig. 1a). An additional model included variables we hypothesized would be associated with PSC-17 scores during the pandemic: caregiver anxiety and depression symptoms, remote assignment completion, change in screen time during the pandemic, knowing someone who became sick with COVID-19, and exposure to COVID-19 media. In this model, we tested the association of these variables with mid-pandemic PSC-17 total scores, while controlling for pre-pandemic PSC-17 total scores and both pre- and mid-pandemic social risk scores.

Full information maximum likelihood estimation was used to handle missing data. Standard indices were used to assess model fit (non-significant Chi Square; Root Mean Square Error of Approximation [RMSEA] < 0.08; Comparative Fit Index [CFI] and Tucker-Lewis Index [TLI] > 0.95) [42].

We used content analysis to code and summarize responses to the open-ended question [43, 44]. First, the research team as a group reviewed the open-ended question responses together and defined an initial set of concepts—or codes—in the data. Then, two authors (AS and MS) independently reviewed the open-ended responses for both presence and frequency of each code, creating new codes as needed when concepts in the text were not encompassed with pre-determined codes. The research team met again to review, discuss and resolve discrepancies in the coding and collapse codes where appropriate to create the final set of codes and frequencies (i.e., unique number of participants whose response included that code).

## Results

### Sample characteristics

We identified 1,051 eligible children from the EHR report, encompassing 913 unique households. We reached 508 households by phone; 223 of the caregivers reached (44%) consented for the study; 168 of the caregivers who consented (75%) answered questionnaire items and were included in the analysis. Overall, there were no significant differences in age, race/ethnicity, or preferred language between the group of all eligible children vs. those who participated. Male children represented a greater proportion of the final sample (57%) vs. the sample of all eligible children (48%), χ^2^(1) = 5.30, *p* < 0.05. There were also no significant differences in pre-pandemic PSC-17 scores (total problems and subscales) between all eligible children vs. participants.

Demographics of the sample are presented in Table 1. Children had a mean age of 8.5 (SD 1.8), were 57% male and 43% female, and majority identifying as Black and Hispanic (54% Non-Hispanic Black, 29% Hispanic, 5% Non-Hispanic White, 2% other, and 10% unknown). Caregiver language preferences were English (79%), Haitian Creole (16%), and Spanish (5%).

**Table 1:** Caregiver and Child Characteristics

	N (%)
Total	168 (100)
Child age	
Mean (SD)	8.5 (1.8)
Child sex	
Male	96 (58)
Female	72 (43)
Child race/ethnicity	
Non-Hispanic Black	91 (54)
Hispanic	48 (29)
Non-Hispanic White	9 (5)
Other	3 (2)
Unknown	17(10)
Caregiver preferred language	
English	132 (79)
Haitian Creole	27 (16)
Spanish	9 (5)
Child insurance	
Public	116 (69)
Commercial	46 (27)
Health Safety-Net/Uninsured	6 (4)
School assignment completion since March 2020	
Little or None	19 (11)
Some	32 (19)
Most or all	111 (66)
Unknown	6 (4)
Change in screen time due to COVID-19	
Much less	6 (4)
Somewhat less	2 (1)
About the same	15 (9)
Somewhat more	36 (21)
Much more	103 (61)
Unknown	6 (4)
Exposure to COVID-19 media	
No	70 (42)
Yes	93 (55)
Unknown	5 (3)
Child knows someone who contracted COVID-19	
No	123 (73)
Yes	40 (24)
Unknown	5 (3)
Caregiver depression (PHQ-2)	
Negative (< 3)	127 (76)
Positive (≥ 3)	33 (20)
Unknown	8 (5)
Caregiver anxiety (GAD-2)	
Negative (< 3)	113 (67)
Positive (≥ 3)	46 (27)
Unknown	9 (5)

*SD* standard deviation, *PHQ-2* Patient Health Questionnaire, 2-item version, *GAD-2* Generalized Anxiety Disorder Screener, 2-item version

### Emotional and behavioral symptoms before and during COVID-19

As shown in Table 2, higher levels of emotional and behavioral symptoms were recorded in the mid-pandemic vs. pre-pandemic period. The mean PSC-17 total score was significantly higher mid-pandemic (*M* = 8.04, *SD* = 6.41) vs. pre-pandemic (*M* = 5.59, *SD* = 5.80), *t*(152) = 6.17, *p* < 0.001, *d* = 0.50, indicating overall worse mental health. Mean scores were also significantly higher on all three subscales of the PSC-17, indicating significantly more attention, internalizing symptoms, and externalizing symptoms mid-pandemic vs. pre-pandemic.

**Table 2:** Child emotional and behavioral symptoms (measured with the PSC-17) and social risks (measured with THRIVE) before and during the COVID-19 pandemic

	Pre-pandemic	Mid-pandemic	Difference testing	Cohen’s *d*
Child emotional and behavioral symptoms				
PSC-17 total problems				
Mean score (SD)	5.59 (5.80)	8.04 (6.41)	*t*(152) = 6.17***	0.50
% positive	8	18	*z* = 2.64**	
PSC-17 internalizing problems				
Mean score (SD)	1.06 (1.56)	2.18 (2.20)	*t*(161) = 6.92***	0.52
% positive	5	18	*z* = 3.72***	
PSC-17 externalizing problems				
Mean score (SD)	1.85 (2.39)	2.58 (2.69)	*t*(160) = 3.99***	0.35
% positive	7	10	*z* = 0.81	
PSC-17 attention problems				
Mean score (SD)	2.68 (2.78)	3.23 (2.64)	*t*(162) = 3.34**	0.37
% positive	14	12	*z* = − 0.63	
THRIVE total score				
Mean (SD)	0.97 (1.60)	2.12 (2.12)	*t*(100) = 6.49***	0.58

Specific social risks	% positive	% positive	*z*-score
Housing insecurity	3	12	2.81**
Food insecurity	16	50	6.74***
Difficulty with transportation	12	14	0.39
Difficulty affording meds	6	9	1.05
Difficulty paying bills	16	38	4.00***
Difficulty with dependent care	1	10	3.27**
Unemployment	3	10	2.55*
Interest in more education	20	39	3.40***

*PSC-17* 17-item Pediatric Symptom Checklist, measuring, *THRIVE* Social Risks Screening Tool

*p < .05, **p < .01, ***p < .001

Similarly, significantly more children had a categorical PSC-17 score indicating overall risk for mental health problems in the mid-pandemic vs. pre-pandemic period (18% vs. 8%, *z* = 2.64, *p* < 0.01). As shown in Table 2, the proportion of children who scored at risk on the internalizing subscale was also significantly greater mid-pandemic vs. pre-pandemic (18% vs. 5%, *z* = 3.72, *p* < 0.001). There were no significant differences in the proportion of children with positive externalizing or attention problems scores between time points.

Almost all children with positive pre-pandemic PSC-17 total scores still had positive mid-pandemic scores (85%), but the majority of children with positive mid-pandemic PSC-17 total scores had screened negative before the pandemic (61%). Among children with positive pre-pandemic PSC-17 total scores (n = 13), scores were not significantly different mid-pandemic vs. pre-pandemic, mean difference = − 0.23, t(12) = − 0.24, p = 0.82, d = 0.06. However, among children who had negative pre-pandemic PSC-17 total scores (n = 139), scores were significantly higher mid-pandemic, mean difference = 2.70, t(138) = 6.44, p < 0.001, d = 0.54.

### Social risks before and during COVID-19

THRIVE social risk data was obtained for the full sample during the mid-pandemic assessment, and was documented in the EHR for 138 families (82%) during the pre-pandemic period. THRIVE scores, indicating the number of caregiver-reported social risks, were significantly higher mid-pandemic (*M* = 2.12, *SD* = 2.12) vs. pre-pandemic (*M* = 0.97, *SD* = 1.60), *t*(100) = 6.49, *p* < 0.001. Table 2 shows the proportion of caregivers reporting each individual social risk before and after the pandemic, including *z*-scores comparing scores by time point. All THRIVE items except difficulties with transportation and affording medication were significantly more prevalent mid- vs. pre-pandemic. Food insecurity increased from 16% of families pre-pandemic to 50% mid-pandemic (*z* = 6.74, *p* < 0.001).

### Social risks and mental health

Pearson correlations for continuous study variables are reported in Table 3. The correlation between pre-pandemic PSC-17 and THRIVE scores (*r* = 0.42, *p* < 0.001) was significantly stronger than the correlation between mid-pandemic PSC-17 and THRIVE scores (*r* = 0.18, *p* < 0.05), *z* = 2.06, *p* < 0.05.

**Table 3:** Correlations between study variables

	1	2	3	4	5	6	7
1. Pre-pandemic PSC-17 total problems	–	–	–	–	–	–	–
2. Mid-pandemic PSC-17 total problems	0.68***	–	–	–	–	–	–
3. Pre-pandemic THRIVE total score	0.42***	0.30**	–	–	–	–	–
4. Mid-pandemic THRIVE total score	0.22**	0.18*	0.59***	–	–	–	–
5. School assignment completion	− 0.37***	− 0.43***	− 0.22*	− 0.10	–	–	–
6. Change in screen time during COVID-19	0.04	0.18*	0.04	− 0.01	− 0.03	–	–
7. Caregiver Depression (PHQ-2)	0.17*	0.37***	0.24*	0.35***	− 0.13	0.09	–
8. Caregiver Anxiety (GAD-2)	0.18*	0.33***	0.27**	0.39***	− 0.16*	0.16*	0.60***

*PSC-17* 17-item Pediatric Symptom Checklist, *THRIVE* Social Risks Screening Tool, *PHQ-2* Patient Health Questionnaire, 2-item version, *GAD-2* Generalized Anxiety Disorder Screener, 2-item version

*p < 0.05, **p < 0.01, ***p < 0.001

We used path analysis to further explore the associations between THRIVE scores and PSC-17 total scores (results shown in Fig. 1b). The model was consistent with good fit, χ^2^(1) = 0.08, *p* = 0.78, RMSEA = 0.00, CFI = 1.00, TLI = 1.04. There was a significant association between THRIVE scores pre- and mid-pandemic (β = 0.57, p < 0.001). There was also a significant association between PSC-17 total scores pre- and mid-pandemic (β = 0.67, p < 0.001). There was a significant relationship between emotional/behavioral symptoms and social risks before the pandemic. Pre-pandemic PSC-17 total scores were significantly correlated with pre-pandemic THRIVE scores (r = 0.39, p < 0.001). However, there was no significant correlation between mid-pandemic PSC-17 total scores and THRIVE scores at either time point. We estimated an additional model to test whether mid-pandemic THRIVE scores moderated the relationship between pre- and mid-pandemic PSC-17 total scores. Although the model was consistent with good fit, the interaction was not significant.

### Additional COVID-related factors and child mental health

We added other mid-pandemic variables to the path model, while continuing to control for pre-pandemic PSC-17 total scores and both pre- and mid-pandemic social risks (Table 4). Children who completed more school assignments had significantly lower mid-pandemic PSC-17 total scores (β = − 0.14, *p* < 0.05), indicating better mental health. Children whose screen time had increased during the pandemic had significantly higher mid-pandemic PSC-17 total scores than children whose screen time stayed the same or decreased (β = 0.15, *p* < 0.01). Caregiver depression symptoms (measured with the PHQ-2) were also significantly associated with increased child mental health symptoms (measured by the PSC-17 total score) mid-pandemic (β = 0.19, *p* < 0.01). Viewing COVID-related media and the extent of child exposure to COVID-related sickness and death were not significantly associated with PSC-17 total scores.

**Table 4:** Path Model: COVID-stressors and mid-pandemic psychiatric symptoms

	Dependent variables	
	Mid-pandemic PSC-17 total problems score	Mid-pandemic THRIVE score
Stressor	β	
Pre-pandemic PSC-17 total problems score	0.57***	–
Pre-pandemic THRIVE score	− 0.01	0.59***
Mid-pandemic THRIVE score	− 0.04	–
School assignment completion	− 0.14*	–
Exposure to COVID-19 media	− 0.10	–
Knowing someone with COVID-19	− 0.05	–
Change in screen time during COVID-19	0.15**	–
Caregiver GAD-2 score	0.07	–
Caregiver PHQ-2 score	0.19**	–

*PSC-17* 17-item Pediatric Symptom Checklist, *THRIVE* Social Risks Screening Tool, *PHQ-2* Patient Health Questionnaire, 2-item version, *GAD-2* Generalized Anxiety Disorder Screener, 2-item version

*p < 0.05, **p < 0.01, ***p < 0.001

Twenty-nine percent of caregivers answered the open-ended question about how COVID-19 impacted their child’s health or well-being (see Additional file 2: Table S1). Caregivers most frequently noted the negative effects of lack of activities outside of the house (n = 11), social isolation (n = 11), fear/anxiety about COVID-19 (n = 7), trouble with remote learning (n = 7), sadness or depression (n = 6), changes in general (n = 5), stress about social determinants (housing, childcare, employment) on the family (n = 5), and lack of exercise or weight gain (n = 4). Five families reported their child was not negatively impacted by the pandemic.

### Assistance with referrals and resources

Forty-five caregivers (27%) asked for assistance with a referral or resource after completing the survey. The most common needs requested included assistance with utility bills (29%), food (27%), housing (18%), financial difficulties (20%), behavioral health referrals (13%), childcare (7%), and education (6%). By partnering closely with the hospital’s Pediatric Integrated Behavioral Health program and Pediatric Resources for COVID-19 program (a.k.a. Project REACH), research staff were able to successfully provide resources for 82% of the needs requested. Examples of specific assistance provided include: connection to an integrated behavioral health clinician, referrals to the hospital food pantry, toy donations, identification of educational programs, free tax assistance, and utility shut-off protection letters for children with chronic illness.

## Discussion

In a sample of predominantly racial and ethnic minority school-age children recruited from an urban primary care setting, we found significantly increased mental health problems—particularly depression and anxiety—during the first year of the COVID-19 pandemic, compared to the prior 6 months. Furthermore, we documented large increases in almost all social risks measured. To our knowledge, this is the first study using pre and mid-pandemic data to quantify the negative psychosocial impact of the COVID-19 pandemic on predominantly racial and ethnic minority urban children.

Internalizing problems (depression and/or anxiety) increased to an alarming 18% in school-age children during the pandemic from 5% prior to the pandemic. Although few studies utilized pre-pandemic data for comparison, research has indicated high levels of depression and anxiety in children during the pandemic, with some evidence that they are connected to pandemic stressors. For example, Xie et al. found that among 1,784 primary school children surveyed in two Chinese cities after 33 days of lock down, 22.6% had symptoms of depression and 18.9% had symptoms of anxiety; these symptoms were significantly associated with worry about COVID [45]. In our qualitative data, caregivers also reported fear about COVID-19 contributing to anxiety.

In our sample, dramatic increases in social risks did not explain the rise in depression and anxiety symptoms. In fact, mental health symptoms were better correlated with social risks *before* the pandemic than *after*. It could be that the mental health impact of social risks is more chronic than acute, and that mental health would be impacted with persistence of these risks [46]. This will be important to measure in the future. In addition, other stressors—such as remote school and social isolation—may have contributed to depression and anxiety in children during the pandemic over and above the contribution of social risks. In the COVID Experiences fall 2020 nationwide survey, caregivers of school-age children were more likely to report worse mental health of children in virtual vs. in-person school (24.9% vs. 15.9%) [47]. Almost all children in our sample were attending school remotely at the time of our mid-pandemic survey, and we found that less remote assignment completion was correlated with increased mental health problems. In the open-ended portion of our survey, parents also reported that trouble with remote learning negatively impacted their child’s health. Therefore, school engagement and success may have a protective role in mental health, and may be an important target of interventions to improve child well-being both during a pandemic as well as in normal times.

We also found a negative association between increased screen time and child mental health. Longstanding concerns about screen time and mental health [48] have become particularly relevant in this time of remote schooling, as many young children have unprecedented access to screens and to the internet. One study suggested that it was not any internet use, but actually problematic internet use (i.e., addictive smart phone, gaming, or internet-related behavior) that was associated with increased psychological stress in children during the pandemic [23]. In addition, most children lacked organized, in-person activities such as sports and extracurriculars during the early phase of COVID-19, which can help deter excessive internet and media usage and also have protective effects for child mental health [49, 50]. In fact, parents reported in our open-ended question that lack of time out of the house, lack of physical activity, and weight gain were important contributors to worse health for their children during the pandemic. On the other hand, parents also described social isolation impacting their child's health during the pandemic, and the internet was an important way to maintain social connections. More research will be needed to understand the COVID-19 pandemic's long term influence on child screen time use, social engagement, participation in activities, and mental health. However, these findings already point to the need for public health efforts to decrease excessive and problematic screen time use in this generation of children, and for planning to prevent excessive screen time as an outcome of future pandemic mitigation measures, while balancing internet use to maintain social connections and prevent isolation.

In addition, we showed a relationship between child mental health and caregiver depression, which is a known risk factor for child mental health problems [51, 52]. Although we cannot assume a causal link, this finding suggests that caregivers’ mental health may have also declined during the pandemic, and this could be an important point of intervention. There is a growing body of literature documenting the negative impacts of the pandemic on caregiver mental health, including a study among 3000 Canadian adults [53] showing worse mental health among parents with minor children at home compared to other adults, and a study showing increased depression, anxiety, and stress among 2365 Australian parents compared to pre-pandemic estimates [54]. This study also found that worse parent functioning was related to pre-existing parent and child physical and mental health conditions, These findings suggest that intervening on the caregiver and family level may be all the more important for children with mental health problems during COVID-19.

Our study has important strengths. This is one of the first studies to include pre-pandemic symptom and social risk data in an examination of child psychosocial functioning during COVID-19. The PSC-17 allowed us to assess symptom severity and identify children at high risk in multiple domains. Enrolling a primary care sample allowed us to show the burden of new mental health concerns in previously asymptomatic children. In addition, we were able to partner with primary care and hospital programs to help address needs that arose in the survey for interested parents. Finally, our sample of urban, predominantly racial and ethnic minority children highlights the psychosocial burden of the pandemic on these disproportionately impacted families.

Our study has a few limitations. We recruited from one hospital in a major city and in just three languages; thus, our findings may not generalize to other geographical locations and populations. Although non-response bias is possible, our sample was not different from the full pool of eligible children in terms of pre-pandemic mental health symptoms. Technological barriers may have prevented some caregivers from participating, although they could complete questionnaires by phone. We also did not have pre-pandemic measures of caregiver mental health, and so it was not possible to measure the effect of changes in caregiver mental health on child mental health during the pandemic. Finally, the impact of the COVID-19 pandemic on child mental health may not be steady over time, and further longitudinal data will be required to understand the long-term implications.

These findings should represent a call to action. Public health efforts to mitigate the psychosocial effects of the pandemic on racial and ethnic minority children and communities will be critical. Pre-pandemic, access to child mental healthcare was already a problem, and this increased volume will be difficult to accommodate within the current mental health system. Rather, public funding sources could support new or expanded community-based, school-based, family-based, and trauma-informed treatment and prevention programs to reach the most affected families. Employing evidence-informed, community health worker approaches may hold particular promise to meet the increased need. Clinical systems and researchers should continue to evaluate pandemic impacts on families over time, as well as the outcomes of publicly funded programs. Continued universal mental health and social needs screening in primary care will be an important part of this ongoing assessment.

## Conclusion

We found increased depression and anxiety problems and social risks during the COVID-19 pandemic compared to before among predominantly racial and ethnic minority school-aged children in an urban pediatric primary care setting. Less school assignment completion, increased screen time, and caregiver depression were significantly associated with increased mid-pandemic mental health symptoms. Additional public health measures will be needed to mitigate this psychosocial crisis for young children.

## Supplementary Information

**Additional file 1.** Mid-pandemic questionnaire.

**Additional file 2.** Content analysis of answers to the open-ended question: “Please tell us anything else you think is important about how coronavirus has impacted your child's health or well-being.

## Data Availability

The datasets used and analysed during the current study are available from the corresponding author on reasonable request.

## Abbreviations

PSC-17: 17-Item Pediatric Symptom Checklist
COVID-19: Coronavirus Disease 2019
THRIVE: Social Needs Screener
EHR: Electronic health record
HIPAA: Health Insurance Portability and Accountability Act
REDCap: Research Electronic Data CAPture
PSC-35: 35-Item Pediatric Symptom Checklist
WE CARE: Well Child Care, Evaluation, Community Resources, Advocacy, Referral, Education
PHQ-2: Patient Health Questionnaire, 2-item version
GAD-2: General Anxiety Disorder Screener, 2-item version
PHQ-9: Patient Health Questionnaire, 9-item version
GAD-7: General Anxiety Disorder Screener, 7-item version
SD: Standard deviation
