# Supplementary material for: Changes in psychosocial functioning among urban, school-age children during the COVID-19 pandemic
Source: Child Adolesc Psychiatry Ment Health. 2021 Dec 2;15:73. doi: 10.1186/s13034-021-00419-w (PMC8637516; doi:10.1186/s13034-021-00419-w)
Supplement: Supplementary file 1 — Additional file 1. Mid-pandemic questionnaire. [file 13034_2021_419_MOESM1_ESM.docx]

**Supplemental File: Mid-pandemic questionnaire**

**SCHOOL**

1. **Did your child continue to participate in remote school after the school closures due to COVID-19 starting in March (*Please select one*)**

- Yes, he/she did **most** or **all** of the assignments
- Yes, he/she did **some** of the assignments
- No, he/she did **little or none** of the assignments
- Other (please specify): _____________________

1. **Did any of the following things make it hard for your child to learn after the school closures in March? (*Check all that apply*)**

- Difficulty with technology
- Less time with teachers
- Less interaction with peers
- Family members unable to help with at-home learning
- Other problems (please specify) _____________________________
- No big problems

**SCREEN TIME**

1. **In the past week, approximately how many hours has your child spent in front of a screen (TV, Computer, IPad/Tablet, Smartphone, etc.) (*Please select one*)**

- None
- Less than one hour
- 1-3 hours
- 3-5 hours
- 5 or more hours

1. **Has COVID-19 changed the amount of time your child spends in front of a screen? (*Please select one*)**

- Much more screen time
- Somewhat more screen time
- About the same amount of screen time
- Somewhat less screen time
- Much less screen time

1. **In the past week, has your child watched the news or viewed other media content related to the COVID-19 (like CNN or videos about the pandemic)? (*Please select one*)**

- Yes
- No

**EXPOSURE TO SICKNESS**

1. **Does your child know anyone who has contracted COVID-19? (*Check all that apply*)**

- No
- Yes, my child knows at least one person who contracted COVID-19

🡪If YES, who?

- - Someone in our household
  - Another family member or close friend (not in our household)
  - Someone else

🡪Also, if YES, how sick was that person?

- - Not hospitalized
  - Hospitalized and recovered
  - Deceased

**Please tell us anything else you think is important about how coronavirus has impacted your child's health or well-being. ___________________________________________________________________________________________________________________________________________________________**
