# Supplementary material for: Changes in psychosocial functioning among urban, school-age children during the COVID-19 pandemic
Source: Child Adolesc Psychiatry Ment Health. 2021 Dec 2;15:73. doi: 10.1186/s13034-021-00419-w (PMC8637516; doi:10.1186/s13034-021-00419-w)
Supplement: Supplementary file 2 — Additional file 2. Content analysis of answers to the open-ended question: “Please tell us anything else you think is important about how coronavirus has impacted your child's health or well-being. [file 13034_2021_419_MOESM2_ESM.docx]

**Table S1.** Content analysis of answers to the open-ended question: “Please tell us anything else you think is important about how coronavirus has impacted your child's health or well-being.”

|  | **N (%)** |
| --- | --- |
| **Total respondents to question** | 49 (100) |
| Lack of activities out of the house | 11 (22) |
| Social Isolation | 11 (22) |
| Fear/anxiety about COVID-19 | 7 (14) |
| Trouble with remote learning | 7 (14) |
| Sadness or depression | 6 (12) |
| Changes in general | 5 (10) |
| Stress about social determinants | 5 (10) |
| No impact | 5 (10) |
| Lack of exercise or weight gain | 4 (8) |
| Family and/or child contracted COVID-19 | 2 (4) |
| Need for PPE/precautions | 2 (4) |
| Too much screen time | 2 (4) |
| Parent worry/stress | 2 (4) |
| Child behavior difficulty | 1 (2) |
